# Deficiency of *MST1* in endometriosis related peritoneal macrophages promoted the autophagy of ectopic endometrial stromal cells by IL-10

**DOI:** 10.3389/fimmu.2022.993788

**Published:** 2022-10-03

**Authors:** Yufei Huang, Shumin Yan, Xiaoyu Dong, Xue Jiao, Shuang Wang, Dong Li, Guoyun Wang

**Affiliations:** ^1^ Department of Obstetrics and Gynecology, Shandong Provincial Hospital, Shandong University, Jinan, Shandong, China; ^2^ Medical Integration and Practice Center, Cheeloo College of Medicine, Shandong University, Jinan, Shandong, China; ^3^ Gynecology Laboratory, Shandong Provincial Hospital, Jinan, Shandong, China; ^4^ Cryomedicine Laboratory, Qilu Hospital of Shandong University, Jinan, Shandong, China

**Keywords:** autophagy, macrophage, endometriosis, *MST1*, *p38-MAPK*, miR-887-5p, IL-10

## Abstract

Changes in the function of peritoneal macrophages contribute to the homeostasis of the peritoneal immune microenvironment in endometriosis. The mechanism by which ectopic tissues escape phagocytic clearance by macrophages to achieve ectopic colonization and proliferation is unknown. The expression of CD163 in peritoneal macrophages in patients with endometriosis is increased, with the overexpression of *MAPK*, which can promote the M2-type polarization of macrophages and reduce their ability to phagocytose ectopic endometrial cells. As an upstream regulator of *MAPK*, *MST1* expression is deficient in peritoneal macrophages of patients with endometriosis. This process is regulated by miR-887-5p, a noncoding RNA targeting *MST1*. Moreover, *MST1*-knockout macrophages secrete anti-inflammatory factor IL-10, which promotes autophagy of ectopic endometrial stromal cells. These results suggest that *MST1* deficient macrophages may accelerate the autophagy of ectopic endometrium *via* IL-10 which was regulated by miR-887-5p.

## Introduction

Endometriosis (EM) is a complicated, chronic inflammatory syndrome (1). It affects nearly 10% of the female population of reproductive age, leading to a sharp decline in quality of life and huge health care costs (2). The clinical symptoms of endometriosis, such as dysmenorrhea, prolonged menstrual period, increased menstrual flow, and painful intercourse place many unnecessary burdens on women’s life and work, which are likely to be evoked by the peripheral and peritoneal immune system and status (3). In addition, infertility and miscarriage due to endometriosis are worse for patients with fertility problems (4–6). In recent years, endometriosis has been defined as a chronic inflammatory disease, and the immune microenvironment of the peritoneal cavity has a bidirectional regulatory effect on the colonization and proliferation of ectopic endometrial tissue, which means that immune dysfunction cannot be ignored in EM causative factors.

Macrophages are essential regulators of innate and adaptive immunity in response to inflammation, damage, repair, and fibrosis in diseases. The recent consensus is that the occurrence and development of EM is inseparable from the local pelvic inflammatory process, and the function of immune cells in the peritoneal environment of EM patients is abnormal (7). The peritoneal macrophages, as tissue-resident macrophages, are the main component of the peritoneal immune microenvironment, and the unusual proportion of peritoneal macrophages of EM have the phagocytosis and abnormal secretion (8–10), but the regulatory mechanism of macrophages in endometriosis remains unknown. Yosuke et al. found that CD206^+^ macrophage (M2-type) is predominated in EM mice (11), and CD163+ macrophage (M2-type) is increased in EM patient (12). Cytokines such as IL-4, IL-10, TGF-β, IL-13 secreted by M2 macrophage have anti-inflammatory effects (13–16), and M2 macrophages are also involved in tissue repair (17, 18). However, the role of polarization changes of EM peritoneal macrophages in the local tissue and organ microenvironment remains unclear.

As a key of the Hippo/YAP pathway,a conserved signaling cascade that mainly regulates abundant biological processes (19), mammalian sterility 20-like kinase 1 (MST1, also known as STK4) controls tissue growth and organogenesis (20). Recent studies have shown that MST1 not only has a major regulatory capacity for embryonic development and tissue remodeling, but also plays a significant role in cancer and benign diseases with cancer biological behavior. Rao et al. found that MST1 binds to Nogo-B to inhibit macrophage M1 polarization and pro-inflammatory factors (21). Downregulation of Hippo/YAP pathway increased apoptosis and decreased the viability of endometriotic cells (22). However, it has not been extensively studied in EM-associated peritoneal macrophages (23). In addition, miRNAs regulate macrophage polarization in different cancers, but the regulatory mechanisms remain unclear (24). We used miRdb (which is a software for predicting microRNAs of target genes, and has a functional annotation database.) to obtain that miRNA-887-5p targets and negatively regulates MST1, and based on this, we hypothesized that miRNA-887-5p-MST1 might mediate the polarization of EM peritoneal macrophages.

The autophagolysosomal degradation system of macroautophagy (hereafter referred to as autophagy) regulates the dynamic balance of diverse pathophysiological conditions, such as metabolic stress, differentiation and development reprogramming, and cell proliferation and migration to safeguard stability (25–28). Autophagy in EM lesions is controversial, and studies have shown that autophagy is activated in EM as manifested by increased LC3II/I ratio and increased Beclin1 expression (29, 30). However, some studies have also found that autophagy is inhibited in ectopic endometrial tissue (31). Regardless, they all suggest that autophagy is closely related to EM.

Whether aberrant autophagy in ectopic endometrial tissue is related to the polarization of peritoneal macrophages and its regulatory mechanism remain unclear. Therefore, the purpose of this study was to explore whether miRNA-887-5p-MST1 of EM peritoneal macrophages affects their polarization, thereby regulating autophagy in ectopic endometrial tissue, and to explore its regulatory mechanism.

## Materials and methods

### Human sample collection

The protocol of this study was approved by the Institutional Review Board of Qilu Hospital of Shandong university (KYLL-2022(ZM)-309). Written informed consent was obtained from all human subjects. We collected peritoneal fluid, eutopic endometrium, and ectopic endometrium from endometriosis patients (mean age 35.51 years; range 24 to 48 years) as the experimental group. The control group (mean age 32.54 years; range 18 to 45 years) comprised non-endometriosis patients (fallopian tube adhesion and simple ovarian cyst), and their peritoneal fluid and eutopic endometrium were collected. There was no statistical difference in the age of patients in the two groups (p = 0.1434). All patients were from Qilu Hospital of Shandong university and were diagnosed by frozen section examination after surgery. None of the patients had received steroid hormone therapy within the past 3–6 months or had pelvic inflammatory disease or related complications, and none exhibited any evident internal medicine or surgical comorbidities (32). Patients in this study were all in the secretory phase of their menstrual cycle, as confirmed by postoperative pathology.

### Human primary cell isolation and culture

During surgical treatment (hysterectomy and laparoscopy), primary peritoneal fluid was collected immediately when the trocar was successfully implanted; thereafter, 100 mL of normal saline was injected into the peritoneal cavity *via* the trocar, and then at least 50 mL of fluid was again collected. Both fluids were pipetted into centrifuge tubes and transferred into a 4°C transport box and immediately transported to the laboratory. The peritoneal fluid was centrifuged (400 x g, for 10 min at 4°C) and cells were resuspended with RPMI 1640 (1875500BT, Gibco, USA) with 10% fetal bovine serum (FBS; 35-081-CV, Corning, USA) and 1% penicillin-streptomycin (PS; 100×) (P1400, Solarbio, USA) at 37°C in a humidified atmosphere containing 5% CO_2_.

We collected eutopic and ectopic endometrium of patients who had undergone hysterectomy and laparoscopic examination. The tissues were gently washed with icy normal saline and PBS three times each, then moved into a small sterile dish and cut into 1 mm^3^ sections with ophthalmic scissors and ophthalmic forceps, followed by incubation with 0.25% (w/v) collagenase II and 0.25% (w/v) collagenase IV for 60 min at 37°C, and then resuspended every 15 min till the tissue-fragments had been thoroughly digested. Collagenase activity was terminated by adding five times volume of prewarmed PBS with 1% FBS. The cell suspension was filtered through 100 μm and 70 μm cell strainers in sequence. After centrifugation at 1000 rpm for 5 min, primary endometrial cells were collected to obtain the precipitates, and then incubated in DMEM (Dulbecco’s modification of Eagle’s medium) (10569044, Gibco, USA) with 10% FBS (Corning) and 1% PS (100×) (Solarbio) at 37°C in a humidified atmosphere containing 5% CO_2_. The medium was changed after 24 h. The purification and culture of endometrial stromal cells were performed by immunofluorescence staining for vimentin and cytokeratin.

### Cell line culture and transfection

HESCs (Human endometrial stromal cells) were cultured in DMEM/f12 (Gibco, Beijing, China), THP-1 cells were also cultured in RPMI 1640 (Gibco, Beijing, China), and both were supplemented with 10% FBS (Corning) and 1% PS (100×) (Solarbio) at 37°C in a humidified atmosphere containing 5% CO_2_. THP-1 cells were transfected with siRNA control and siRNA-*MST1* (BoShang, Shanghai, China) using Lipofectamine RNAiMAX Transfection Reagent (13778150, Invitrogen, USA). Lipofectamine 3000 Transfection Reagent (L3000015, Invitrogen, USA) was used to transfect control mimics, miR-887-5p mimics, inhibitor control, and miR-887-5p inhibitor (GenePharma, Shanghai, China), according to the manufacturer’s instructions. For knockdown of *MST1*, sequences targeting *MST1* were designed and synthesized by GenePharma Co., and the culture medium was replaced after 12 h of incubation. Some of the transfected cells were incubated on glass slides. After 36 h of transfection, cells were collected for subsequent experiments, such as RNA extraction, RT-qPCR, protein extraction, western blotting, cell immunofluorescence, and flow cytometry.

For co-culture, HESCs, used as the local cell line, were seeded in the lower chamber of a 24-well plate. Next, si*MST1*, miR-887-5p mimics, and miR-887-5p inhibitor (and the negative control (NC) mimics or inhibitor) were transfected into PMA (Phorbol 12-Myristate 13-Acetate)-treated THP-1 cells, respectively, after which 2 × 10^5^ cells were seeded in a cell insert (Corning, NY, USA) with a pore size of 0.3 µm in the upper chamber. THP-1 cells and HESCs were at a 2:1 ratio for 48 h. During the whole co-culture, the HESCs at the bottom of the 24-well plate could not directly contact the THP-1 cells at the top chamber. HESCs were then collected for other experiments.

### Macrophage polarization

THP-1 (1 × 10^6^ cells/mL) were seeded in a 24-well plate and cultured for 48 h in RPMI (Roswell Park Memorial Institute) 1640 medium with PMA (200 nM). The M0-only phenotype was created by adding PMA; after 48 h, the M0 macrophages were polarized into M1 macrophages by adding LPS (100 ng/mL) for another 24 h, or into M2 macrophages by adding human-IL-4 (20 ng/mL) for another 24 h.

### RNA extraction and RT-qPCR

Using TRIzol (15596-026, LIFE Ambion TRIzol, USA) reagent, we extracted the total RNA from tissues, and then 1 μg RNA was reverse-transcribed into cDNA using a reverse transcription kit, and the target gene fragment was amplified and detected with a SYBR Green qPCR kit (TOYOBO, QPK-201, Japan). RT-qPCR was performed for 40 cycles. For reverse transcription, total RNA was prepared from cells using TRIzol reagent according to the manufacturer’s instructions (Invitrogen, Waltham, MA, USA). QuickEasy™ Cell Direct RT-qPCR Kit-SYBR Green (DRT-01012, FOREGENE, China) was used to directly lyse cells to release RNA for RT-qPCR. We analyzed the RT-PCR result of peritoneal macrophages from EM group(n=10) and non-EM group(n=10), and of the tissues from EM-group including eutopic endometrium and ectopic endometrium (n=10), and non-EM group including eutopic endometrium(n=10). miRNA levels were normalized to those of U6 in cells, and mRNA expression was normalized to that of *GAPDH*. The 2^−ΔΔCt^ method was used for quantification. All experiments were performed three times, at least for each sample. Relative gene expression was analyzed using the 2^−ΔΔCT^ method.

### Total protein extraction and western blot

The collected tissues were divided into several pieces, each weighing 50 mg, and were washed three times with precooled 4°C PBS, triturated with 20 mL liquid nitrogen, and then 500 μL RIPA buffer with 1% PMSF (Phenylmethylsulfonyl fluoride) on ice was added. Cells were washed three times with PBS, and then 250 μL RIPA buffer with 1% PMSF was added per 1 × 10^6^ cells, after which the mixture was pipetted into a 1.5 mL tube and incubated for 30 min on ice before being centrifuged at 12 000 rpm at 4°C for 15 min. The concentration of proteins (40 μg per sample) was then measured, and the protein supernatant with 5× SDS-loading buffer was warmed in a metal bath at 100°C for 10 min. Each protein sample was separated using 12.5% gel from the ExpressCast PAGE Preparation kit (P2013, New Cell & Molecular Biotech, China) for approximately 1 h and then transferred onto PVDF (polyvinylidene fluoride) (0.22 μm) membranes for 30 min by Rapid Transfer Buffer (WB4600, New Cell & Molecular Biotech) and blocked with Rapid Block Buffer (P30500, New Cell & Molecular Biotech) for 20 min at about 26°C. Afterwards, the PVDF membranes were placed in diluted primary antibody solutions (diluted by Western Primary Antibody Dilution Buffer, P0023A, Beyotime, China) of *MST1* (ab110240, Abcam, UK; 1:10 000), *p38-MAPK* (ab170099, Abcam, UK; 1:1000), *Beclin1* (ab62557, Abcam, UK; 1:1000), *LC3B* (ab51520, Abcam, USA; 1:1000), *SQSTM1/p62* (ab109012, Abcam, USA; 1:1000), and GAPDH (AB-P-R001, GoodHere, China; 1:1000) at 4°C overnight, and then incubated with diluted secondary antibodies (diluted by (Tris Buffered Saline with Tween^®^ 20)) of HRP-labelled Goat Anti-Mouse IgG (H+L) (A0216, Beyotime, China; 1:10 000) and HRP-labelled Goat Anti-Rabbit IgG (H+L) (A0208, Beyotime, China; 1:10 000) for 90 min, and expression was detected by an HRP chemiluminescence detection kit (WBKLS0100, MILLIPORE Immobilon TM Western Chemiluminescent HRP Substrate, USA). Each experiment was repeated at least three times.

### Autophagy flux detection

HESCs and primary endometrial cells (including endometriosis eutopic, ectopic endometrial cells, and control ectopic endometrial cells) were cultured in DMEM/F12 supplemented with 10% FBS (Gibco) and PS (Gibco) at 37°C in a humidified atmosphere of 5% CO_2_. All cells were cultured on a 24-well plate (Corning) containing microscope cover glass slips (WHB, Shanghai, China). Cells were infected with mCherry-GFP-*LC3* adenovirus overnight, the medium was changed, and cells were cultured for 72 h. Next, we added the transfected-THP-1 (treated with si*MST1*, miR-887-5p mimics, and miR-887-5p inhibitor) cells into a cell culture insert with a pore size of 0.3 µm, and co-cultured them with HESCs for 24 h. Cells were covered with 100 µL Cytofix/Cytoperm solution (BD) for 20 min at 4°C, and then washed two times in 1× Perm/Wash solution (diluted with cold PBS). We observed the co-localized point clusters of red/green and acquired images using the 63× oil lens of a laser confocal microscope (LSM880 Airy, Zeiss), illuminating with a 488 nm multi-Ar laser (GFP fluorochrome excitation) or with a 561/595 nm diode-pumped solid-state laser (mCherry fluorochrome excitation). GFP-*LC3* (green dots), autophagosomes (yellow dots), and autolysosome (red dots) were detected. Images were processed with the Zen software (Zeiss).

### Immunofluorescence staining

Primary HESCs, human peritoneal macrophages, THP-1 cells, and HESCs after multiple treatments were fixed in ice-cold methanol for 15 min, washed three times with 4°C PBS, incubated for 15 min in 0.25% Triton X-100 diluted in PBS, immersed in PBS twice for 3 min, and blocked with 1% BSA in PBS for 30 min at about 26°C. The primary HESCs and HESCs were incubated with anti-*Beclin1* (1:100; ab62557, Abcam), anti-*LC3B* (1:100; ab51520, Abcam), *SQSTM1/p62* (1:500; ab109012, Abcam), and *ULK1* (1:200; ab203207, Abcam). Human peritoneal macrophages and THP-1 cells were incubated with anti-*MST1* (1:400; ab51134, Abcam) and anti-*p38-MAPK* (1:200; ab170099, Abcam). The incubation time of each antibody varied from 1 h to 24 h at 4°C. All antibodies were diluted in 1% BSA in PBS. After cells were immersed in PBS twice for 3 min, they were incubated with the anti-mouse/rabbit secondary antibody for 30 min at about 26°C. Anti-quench nuclear staining and mounting with DAPI (ab104139, Abcam, USA) were performed. Images were captured with a confocal microscope (LSM880 Airy, Zeiss) and processed with the Zen software (Zeiss).

### FCM (Flow cytometry) analysis

The cells collected from the peritoneal fluid after 24 h of culture for adherence were fixed with a buffer containing paraformaldehyde (554722, BD Cytofix/Cytoperm USA), washed two times with 1× BD wash buffer (554723, BD Perm/Wash, USA), centrifuged at 350 × *g* for 5 min, and then the supernatant was discarded. For membrane cytokines, such as CD163 (anti-human CD163; 333606, BioLegend, USA) and CD86 (anti-human CD86; 374216, BioLegend, USA), cells were incubated in diluted primary antibody buffer in the dark for 20 min at about 26°C. For intracellular cytokines, such as CD68 (anti-human CD68; 333806, BioLegend, USA) perm buffer (554723, BD Perm/Wash, USA) was used for 15 min for permeabilization, and the supernatant was discarded after centrifuging at 350 × *g* for 5 min. Cells were incubated in diluted primary antibody buffer in the dark for 30 min at 4 °C, washed twice with wash buffer, and centrifuged at 350 × *g* for 5 min. The cells were then resuspended in perm buffer for FCM analysis.

### CCK-8 assay

We used a CCK-8 kit (BestBio, China, BB-4202-500T) to measure the proliferation of HESCs after co-culture. Briefly, 1 × 10^4^ cells in 100 μL of medium were seeded per well in a 96-well plate according to the manufacturer’s protocol, with three replicate wells. Next, the CCK-8 reagent (10 μL) was added to 90 μL DMEM/f12 to prepare the working fluid followed by incubation with the cells for 24 h for the best results. The proliferation of cells was described by the absorbance.

### Dual-luciferase reporter gene assay

The sequence of miR-887-5p mutants was cloned and inserted into the 3′UTR of the *MST1* (STK4) plasmid (Searching Biotechnology). In a 6-well plate, 293T cells were cultured to approximately 70% confluence and then co-transfected with either WT or mut luciferase reporter vector (2 μg) and either mimic miRNAs or NC (2 μg). After 48 h, luciferase activity was measured and normalized to the activity of Rluc.

### IL-10 concentration of ELISA kit

We collected all the cell culture media which used to detect the concentration of IL-10, centrifuged the samples at 2-8°C and 900× *g* for 15min, and collected the supernatant; according to the kit instructions (Mouse IL-10 Uncoated ELISA, Invitrogen, 88- 7105; Human IL-10 ELISA kit, Multi Sciences, EK110/2-96) followed by operations, terminated the reaction and read plate at 450 nm. We made a standard curve according to the standard concentration and OD value, and calculated the concentration of the sample to be tested according to the standard curve equation.

### Statistical analysis

Statistical analyses were performed using SPSS IBM 20.0. Statistical significance was determined by the t-test or ANOVA and the Mann–Whitney test (P < 0.05). Kaplan–Meier survival analysis and log-rank tests were used to analyze the difference in survival.

## Results

### CD68^+^CD163^+^ macrophages dominate the peritoneal microenvironment in endometriosis

**Figure 1:**
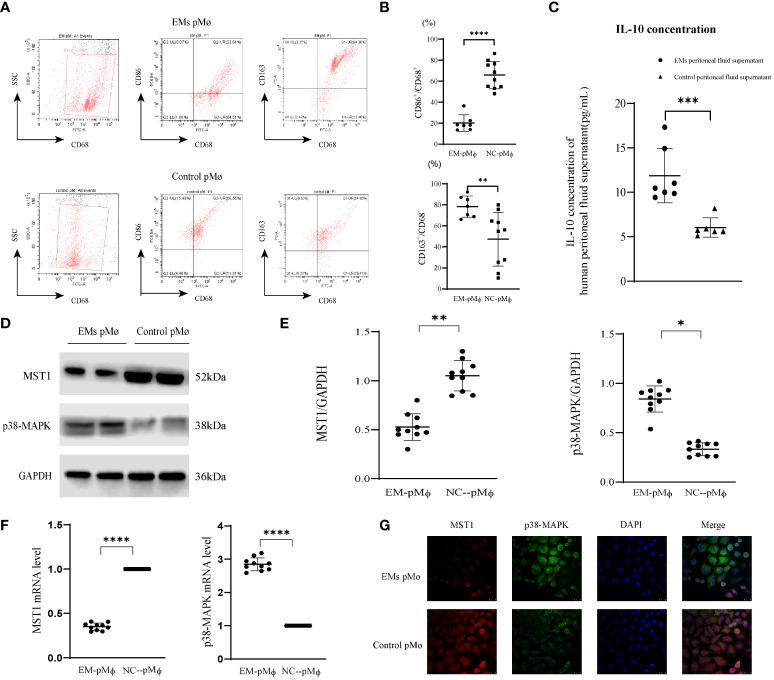
*MST1* is downregulated in endometriosis-related peritoneal macrophages. **(A, B)** Intraperitoneal cells were collected from patients with or without endometriosis and then analyzed by flow cytometry. A higher percentage of CD68^+^CD86^+^ macrophages was present in the peritoneal cavity of the control group, while the percentage of CD68^+^CD163^+^ macrophages was higher in the peritoneal cavity of endometriosis patients. **(C)** The IL-10 concentration of human peritoneal fluid supernatant with or without endometriosis. **(D, E)** Representative western blot of protein expression of *MST1*, and *p38-MAPK*. **(F)** The mRNA level of *MST1* and *p38-MAPK* by RT-qPCR. **(G)** The location of *MST1* and *p38-MAPK* by immunofluorescence staining. *P < 0.05, **P < 0.01, ***P < 0.001, ****P < 0.0001 (Control group peritoneal macrophages vs. endometriosis group peritoneal macrophages). Values represent the mean ± standard error.

We extracted peritoneal fluid from patients with endometriosis and non-endometriosis and obtained peritoneal cells after centrifugation. Flow cytometric analysis showed that the abundance of CD68^+^CD86^+^ cells was lower than that of CD68^+^CD163^+^ in endometriosis-related peritoneal cells (**Figures 1A, B**). Previous studies have found that the anti-inflammatory factor IL-10 is a marker of M2 macrophages (33), we examined the secretion of IL-10 in the peritoneal fluid of EM patients and found the higher concentration of IL-10 compared with the control group (**Figure 1C**). These data mean the M2-type macrophages predominate among of peritoneal macrophages in endometriosis, and have a high level of IL-10 secretion.

### *MST1* is downregulated in endometriosis peritoneal macrophages

*MST1* in macrophages assists in the regulation of oxidative stress and autophagy and is closely related to immune regulation; however, its role in peritoneal macrophages has not yet been studied. Therefore, we detected the expression of *MST1* in peritoneal macrophages in patients with endometriosis and found that its protein and mRNA levels were decreased (**Figures 1D–F**). The regulatory factors of *MST1* transcription may be involved in the abnormal expression of *MST1*. For macrophage polarization, *p38-MAPK* is not only downstream of *MST1* but also a positive regulator of M2 macrophages (34). We detected that the expression of *p38-MAPK* was increased in the peritoneal macrophages of endometriosis (**Figures 1D–F**). For visual observation, we examined *MST1* and *p38-MAPK* colocalization, and found that *MST1* and *p38-MAPK* were localized in the cytoplasm (**Figure 1G**). As an important molecule of the HIPPO pathway, *MST1* plays an important role in tissue development and differentiation, cell proliferation, and apoptosis. *MST1* is the upstream-negative regulatory molecule of *p38-MAPK*, which may have an important regulatory role in gastric cancer and other malignant tumors and certain organogenesis; however, research on the pathogenesis of endometriosis is rarely reported.

### miRNA-887-5p expression increased in endometriosis-related peritoneal macrophages

**Figure 2:**
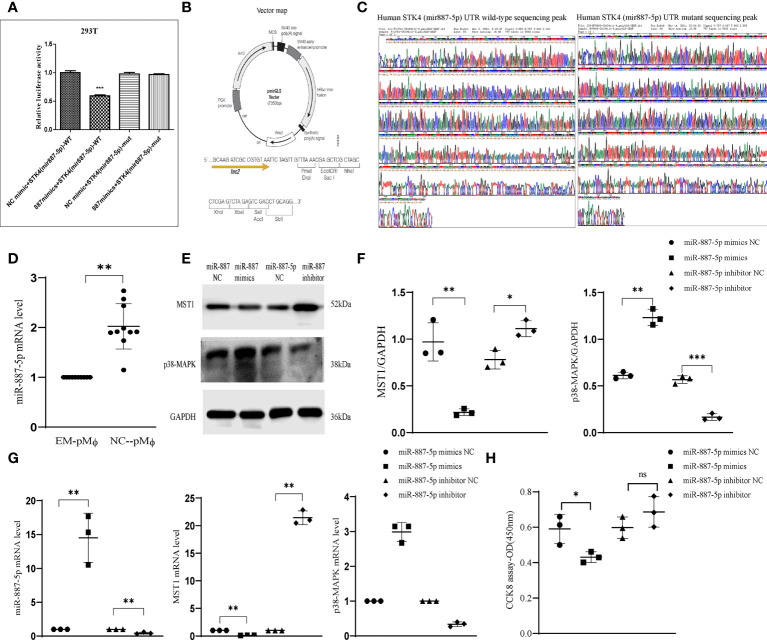
miRNA-887-5p increased in peritoneal macrophages of endometriosis. **(A)** Dual-luciferase reporter assay was conducted in 293T cells co-transfected with miR-NC or miR-887-5p. **(B)** Vector construction for luciferase experiment. **(C)** *MST1*(STK4)-miR-887-5p UTR sequencing peak. **(D)** The mRNA levels of miR-887-5p in human peritoneal macrophages with or without endometriosis by RT-qPCR. **(E, F)** Representative western blot of protein expression after transfection. **(G)** The mRNA level of *MST1* and *p38-MAPK* after miR-887-mimics and -inhibitor transfection. **(H)** The cell viability of THP-1 after transfection. *P < 0.05, **P < 0.01, ***P < 0.001 (Control group peritoneal macrophages vs. endometriosis group peritoneal macrophages). Values represent the mean ± standard error. ns means not statistically significant.

Because a similar trend in expression differences of the mRNA and protein levels of *MST1* in peritoneal macrophages of patients with endometriosis suggests differences in gene transcription modification levels, we used software (TargetScan, miRcode, and PicTar) to predict miRNAs related to *MST1* and obtained several of them. The 3′UTR binding verification was conducted, and miR-887-5p was screened out as the target miRNA that binds to *MST1*. We constructed a luciferase reporter plasmid assay (STK4-wild type (WT), STK4-mutant (mut)) based on the predicted miR-887-5p binding site. The results showed that luciferase activity was quenched in 293T cells, which were co-transfected with miR-887-5p mimics and *MST1* (STK4)-WT, indicating that *MST1* could bind to the 3′UTR of miR-887-5p (**Figures 2A–C**). We then used quantitative real-time PCR (RT-qPCR) to detect the expression of miR-887-5p in peritoneal macrophages of patients with endometriosis and observed that it was increased (**Figure 2D**). With the increase in miR-887-5p expression through being transfected with mimics-miR-887-5p in THP-1 cells (human myeloid leukemia mononuclear cells), the expression of *MST1* decreased and that of *p38-MAPK* increased. Silencing of miR-887-5p by the transfected inhibitor activated the expression of *MST1* and inhibited the expression of *p38-MAPK* (**Figures 2E–G**). And the cell viability is inhibited with a higher level of miR-887-5p in THP-1 (Figure 2H), while miR-887-5p-inhibitor-THP-1 has a normal cell viability. The negative regulation of *MST1* by miR-887-5p could be one reason for the abnormal peritoneal macrophages in endometriosis.

### Autophagy may be increased in endometriosis ectopic endometrium

**Figure 3:**
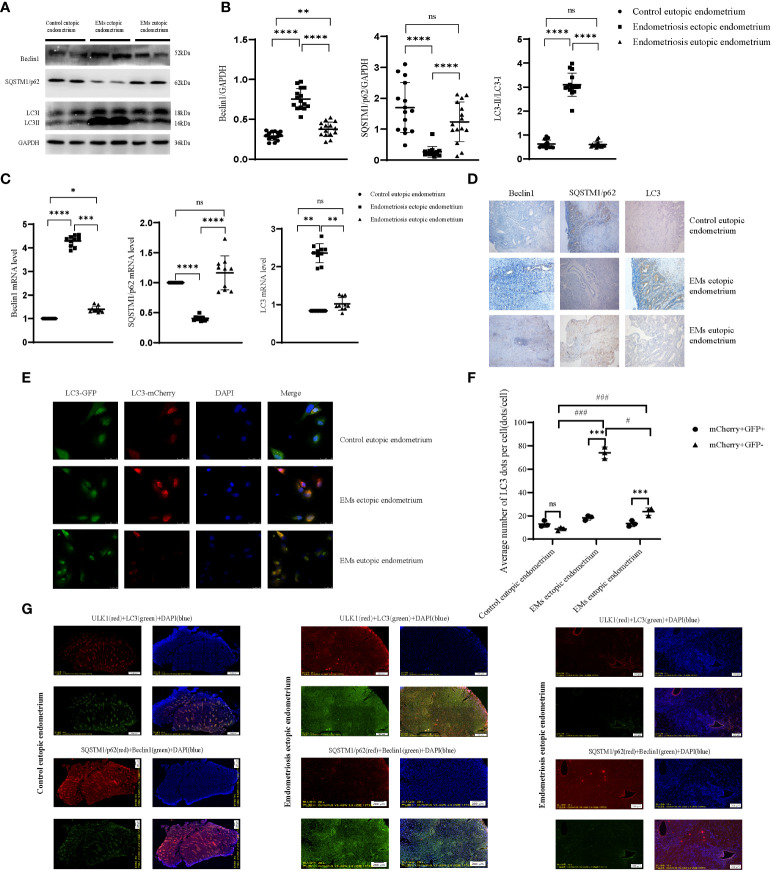
Autophagy is anomalous in endometriosis ectopic endometrium. **(A, B)** The protein level of autophagic genes by western blot; representative examples of *Beclin1*, *SQSTM1/p62*, and *LC3B*. **(C)** The mRNA level of autophagic genes. **(D)** Representative examples of immunohistochemistry staining of *Beclin1*, *SQSTM1/p62*, and *LC3B* in the endometrial tissues of endometriosis eutopic and ectopic endometrium (vs control eutopic endometrium). **(E, F)** The human primary endometrial cells were transduced with mCherry-GFP-*LC3* adenovirus to evaluate the autophagy flux; the yellow dots indicate autophagosomes, while the red dots indicate autolysosomes. At least 5–10 cells per condition were imaged by a confocal microscope. **(G)** Representative image of the fluorescence of panoramic paraffin tissue slices with the autophagic gene stain, including ectopic and eutopic endometrium of endometriosis (vs control eutopic endometrium); each tissue wax block was continuously sliced, choosing two adjacent slices that have been labelled with autophagy-associated proteins (*Beclin1*, *LC3*, *SQSTM1/p62*, *ULK1*). *P < 0.05, **P < 0.01, ***P < 0.001, ****P < 0.0001, ^#^P < 0.05, ^###^P < 0.001, ns means not statistically significant. Values represent the mean ± standard error.

*Beclin1* and *LC3II/I* levels of the ectopic endometrium of patients with endometriosis were higher than those of the eutopic endometrium and self-eutopic endometrium of normal patients. However, *SQSTM1/p62* depletion was higher, with less accumulation. Multiplex immunofluorescence staining showed that *Beclin1* expression was concentrated in the cytoplasm, especially in the Golgi apparatus. *LC3B* was localized in the cytoplasm and the endomembrane system, and specifically, *LC3*-II bound to the autophagosome membrane. Autophagic membranes and p62/SQSTM were concentrated in the cytoplasm, but with a weak fluorescence signal. *ULK1* regulated the formation of autophagophores, the precursors of autophagosomes, and may phosphorylate *SQSTM1/p62*, which is in the cytoplasm, to regulate autophagy (**Figure 3G**). To further clarify the abnormal expression of autophagy-related proteins in endometriotic lesions, we used western blotting to semi-quantitate their protein expression (**Figures 3A–C**). The expression of *Beclin1* was significantly increased, the ratio of *LC3II/I* was increased, and the expression of *SQSTM1/p62* was decreased, which indicated that autophagy was activated and its level was anormal in the ectopic lesions of patients with endometriosis. Similar to malignant tumors, this level of autophagy is likely to contribute to another aspect of the biological behavior of many malignant tumor cells in the endometrial tissue of patients with endometriosis. Immunohistochemistry results corresponded with these results (**Figure 3D**). To evaluate the intensity of the autophagy process in human primary endometriosis ectopic (Figures 3E, F) endometrial stromal cells, we injected the GFP-mCherry-*LC3* (**Figures 3E, F**) adenovirus into primary endometrial stromal cells (eutopic and/or ectopic) isolated from patients with and without endometriosis. The GFP fluorescence signal weakened and the mCherry fluorescence signal was enhanced, suggesting that lysosomes and autophagosomes fused to form autophagolysosomes in ectopic endometrial stromal cells of patients with endometriosis (vs. eutopic endometrial cells of patients with endometriosis and non-endometriosis patients), combined with decreased p62 expression, suggesting that autophagic lysosomes and the body degradation pathway are activated, and the level of autophagy increases (**Figure 3F**). These results imply that the autophagy level of the ectopic endometrium of patients with endometriosis may be increased, and it is strongly suggestive that high autophagy might provide energy and maintain colonization and proliferation of endometriotic cells. To further understand the relationship between abnormal autophagy in endometriosis and ectopic endometrial tissue and the regulation of peritoneal macrophages, we performed a series of *in vitro* and *in vivo* experiments.

### *MST1*-*p38-MAPK* regulated the function of macrophages

**Figure 4:**
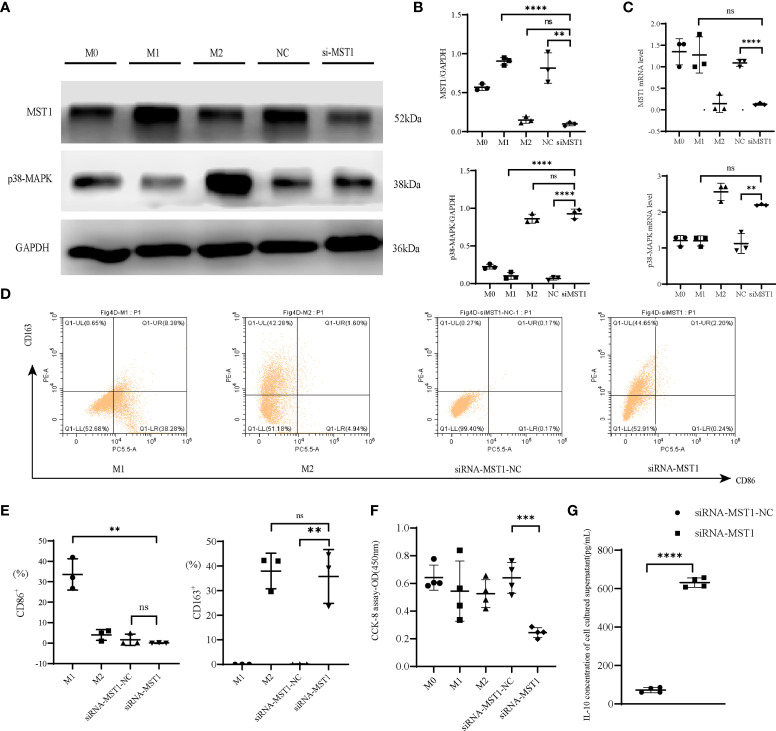
*MST1*-p38*MAPK* regulated the function of macrophages. **(A, B)** Representative western blot and relative protein expression of *MST1* and *p38-MAPK* after transfecting cells with siRNA-*MST1*. **(C)** The mRNA level of *MST1* and *p38-MAPK* after transfecting cells with siRNA-*MST1* by RT-PCR. **(D, E)** Representative flow cytometry to evaluate the polarization state when the expression of *MST1* was decreased. **(F)** Cell viability after transfection. **(G)** The IL-10 concentration of the culture supernatant after transfection of siRNA-*MST1* in THP-1. **P < 0.01, ***P < 0.001, ****P < 0.0001, ns means not statistically significant. Values represent the mean ± standard error.

To explore the effect of *MST1* and *p38-MAPK* on macrophage function in immunity, we used THP-1 cells for vitro experiments. We used siRNA-*MST1* to knock down the expression of *MST1* in THP-1. With the deficiency of *MST1*, *p38-MAPK* accumulated (**Figures 4A–C**). We detected the polarization of them, we found that the siRNA-*MST1*-THP-1 overexpress CD163, same to M2-type. (**Figures 4D, E**). similar to siRNA-*MST1*, M2-type had a lower level of *MST1*, while M1-type had a higher level of *MST1*. Meanwhile, the expression of *p38-MAPK* was negatively correlated with *MST1*. Similar to siRNA-*MST1*, the expression of *p38-MAPK* of M2-type THP-1 is increased, while that of M1-type THP-1 is decreased. THP-1 with a lower level of *MST1* had a negative influence on macrophage viability, but the cell viability of M2-type-THP-1 was not inhibited (**Figure 4F**). Meanwhile, the IL-10 concentration of siRNA-*MST1*-THP-1 was increased (**Figure 4G**). This is consistent with the biological properties of peritoneal macrophages in patients with endometriosis.

### *MST1* knock out in macrophages induced human endometrial stromal cell (HESC) autophagy

**Figure 5:**
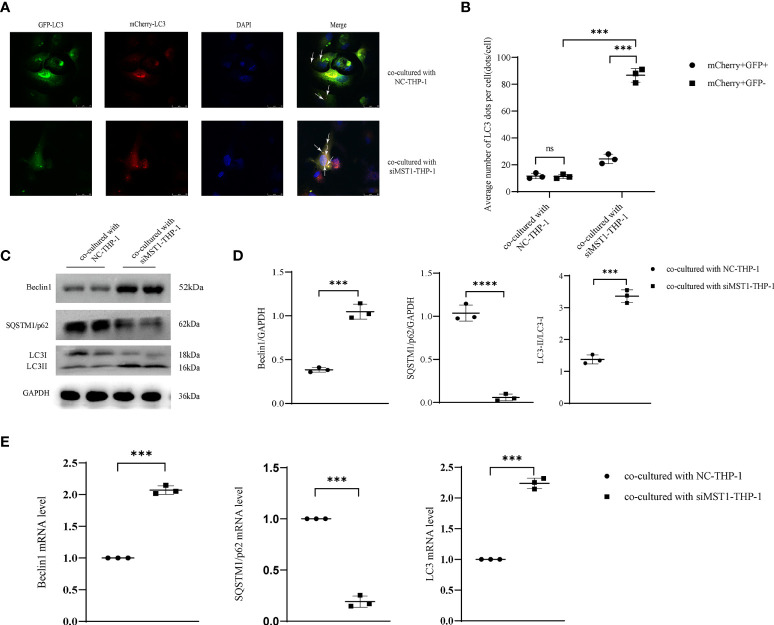
*MST1* knockout from macrophages induced HESC autophagy by co-culture. **(A, B)** HESCs co-cultured with siRNA-*MST1*-THP-1, and then mCherry-GFP-*LC3* adenovirus was transduced into HESCs to evaluate the autophagy flux. **(C, D)** The representative images of protein expression of autophagic genes in HESCs co-cultured with siRNA-*MST1*-THP-1 by western blot. **(E)** The mRNA level of autophagic genes in HESCs co-cultured with siRNA-*MST1*-THP-1 by RT-PCR. ***P < 0.001, ****P < 0.0001, ns means not statistically significant. Values represent the mean ± standard error.

To explore whether the expression of *MST1* in macrophages could affect the autophagy in endometrial cells, we co-cultured THP-1 cells transfected with si*MST1* and HESCs. After co-culture, HESCs were transfected with GFP-mCherry-*LC3* adenovirus, and the autophagy flux was detected by confocal fluorescence microscopy. The results showed that autophagosome and lysosome fusion caused the appearance of red spots, and the absence of yellow spots indicated an increase in autophagy flux (**Figures 5A, B**). The expression of several key autophagic proteins was also evaluated. Western blot analysis showed that *LC3II/I* and *Beclin1* levels were increased, whereas *SQSTM1/p62* expression was decreased (**Figures 5C, D**). The change in the mRNA levels of these autophagic genes was similar to that of proteins (**Figure 5E**). Overall, macrophages with a low expression of *MST1* activated autophagy in endometrial cells by co-culture, which might be a way to increase the survival of ectopic endometrial cells.

### miR-887-5p inhibits the expression of *MST1* in THP-1 cells and activates autophagy in HESCs *via* IL-10

**Figure 6:**
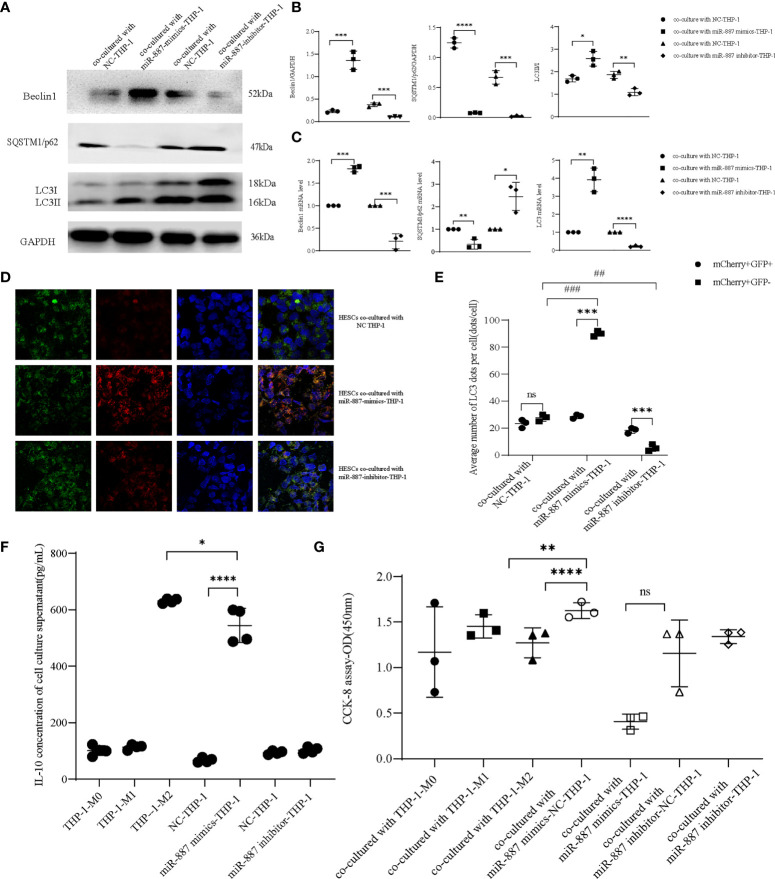
miR-887-5p inhibits the expression of *MST1* in THP-1 cells and activates autophagy in HESCs. **(A, B)** HESCs co-cultured with miR-887-5p-mimics and/or -inhibitor-THP-1, the protein expression of autophagic genes by western blot. **(C)** The mRNA level of autophagic genes in co-cultured HESCs by RT-PCR. **(D, E)** HESCs co-cultured with miR-887-5p-mimics and/or -inhibitor-THP-1, then were transduced with mCherry-GFP-*LC3* adenovirus. At least 10–20 cells per condition were imaged using a confocal microscope. **(F)** The IL-10 concentration of the culture supernatant after transfection of miR-887-5p-mimics and -inhibitor in THP-1 **(G)** The cell viability of co-cultured HESCs. *P < 0.05, **P < 0.01, ***P < 0.001, ****P < 0.0001, ^##^P < 0.01, ^###^P < 0.001, ns means not statistically significant. Values represent the mean ± standard error.

THP-1 cells were transfected with miR-887-5p mimics, then we co-cultured them with HESCs. This promoted autophagy in HESCs; the protein and mRNA levels of *Beclin1* and *LC3II/I* were increased, while those of *SQSTM1/p62* decreased, indicating that overexpression of miR-887-5p in THP-1 cells induced autophagy in co-cultured HESCs. Transfection of cells with the miR-887-5p inhibitor showed the opposite result, validating the negative regulatory effect of miR-887-5p on *MST1* (**Figures 6A–C**). Furthermore, overexpression of miR-887-5p downregulated *MST1* in THP-1 cells, reduced the autophagic flux, and blocked autophagy (**Figures 6D, E**). Based on the above results, we assumed that *MST1* ^low^ macrophages may be the inducers of abnormal autophagy in ectopic endometrial cells. IL-10 secretion is increased in the synovial fluid of Rheumatoid arthritis (RA), accompanied by abnormal neutrophil autophagy (35). In order to assess the secretion of IL-10 in *MST1* ^low^ macrophages and the effect on autophagy of ectopic endometrial cells, we collected the culture supernatant of macrophages which were transfected with miR-887-5p-mimics and -inhibitor, then we performed Elisa to detect IL-10 secretion, and found that macrophages after upregulating miR-887-5p have a higher level of IL-10 secretion (**Figure 6F**), which is similar to siRNA-*MST1*-THP-1. The inhibition of *MST1* by transfecting miR-887-5p mimics decreased the viability of HESCs after co-culture (**Figure 6G**). Therefore, *MST1* ^low^ macrophages secrete IL-10 excessively, which may induce autophagy in endometrial cells.

## Discussion

In this study, we found reduced mRNA expression of *MST1* (STK4) in peritoneal macrophages of patients with endometriosis, whereas the expression of miR-887-5p as an upstream regulator of *MST1* was increased. CD68^+^ peritoneal macrophages (pMφ) co-express *MST1* with low expression in endometriosis. The peritoneal macrophages of endometriosis patients, the pMφ of patients with endometriosis with high CD206 expression, and the low expression of CD86 suggest that the pMφ of patients with endometriosis was skewed towards the M2 phenotype. We knocked down *MST1* in macrophages and detected increased expression of *p38-MAPK*, and macrophages were skewed towards the M2 phenotype as an anti-inflammatory factor. In addition, macrophages with *MST1* knockdown co-cultured with HESCs activated autophagy in HESCs, and this phenomenon coincided with the autophagy activation state in the ectopic endometrial cells of endometriosis patients.

*MST1* is a group II germinal center kinase that acts as an upstream effector in the Hippo pathway, and it can be autophosphorylated. Considering that *MST1* can be spatially regulated by membrane targeting, intracellular transport based on the cytoskeleton may play a key role in regulating this cascade (36), which may be the signal transmission mechanism. The Hippo pathway is widely dysregulated, especially by *MST1*, in multiple cancers, including glioma, colorectal cancer, and endometrial cancer, demonstrating that inactivation of *MST1* inhibited cancer cell growth and apoptosis (37, 38). However, the regulatory mechanism of *MST1* in endometriosis, especially endometriosis-related macrophages, is unknown. The expression of active YAP due to *MST1*/2 knockout in mouse liver induces the recruitment of M2-type macrophages by cytokines, resulting in the establishment of an immunosuppressive microenvironment (39). Genetic ablation of *MST1* in bone marrow-derived macrophages in mice reinforced the expression of NF-κB target genes (40). These results on *MST1* provide a better insight into the control of immune homeostasis. In summary, *MST1* could maintain immune homeostasis, and inhibition of *MST1* may curb disease progression and purge the disease-related inflammation environment. We presume that *MST1* may also regulate the peritoneal immune macroenvironment in endometriosis. When we collected peritoneal fluid from patients with endometriosis and separated monocytes from it and then assessed the monocytes by flow cytometry, the CD68^+^CD206^+^ cell cluster occupied a major position in the peritoneal immune cells of endometriosis patients (compared with non-endometriosis patients). Zou et al. (41). found that immune cells in the peritoneum are mainly responsible for the clearance of refluxed endometrial debris and alleviating the pro-inflammatory effect to repair tissue. *In vivo*, the conventional polarization type of a pro-inflammatory/pro-repair model could not present peritoneal macrophage heterogeneity (42, 43). Macrophages lacking p38 underwent M2 polarization and were marked as CD45^+^F4/80^+^CD11b^+^CD206^+^, which is similar to the observation in peritoneal macrophages from endometriosis patients marked as CD68^+^CD206^+^. Moreover, we detected lower protein and mRNA levels of *MST1* and higher levels of *p38-MAPK* in peritoneal macrophages from patients with endometriosis (vs. non-endometriosis). Cell immunofluorescence showed similar results, as *MST1* and *p38-MAPK* were in the cytoplasm and nucleus. The inhibition of *MST1* in macrophages induced by M2-type macrophages was similar to that of endometriosis-related peritoneal macrophages. Finally, we transfected the mimics and/or inhibited miR-887-5p in THP-1 cells after stimulation with PMA and found that miR-887-5p was a negative regulator of *MST1*, helping to accumulate *p38-MAPK* and also inducing the polarization of M2-type macrophages. Owing to the anomalous type of peritoneal macrophages in endometriosis patients, the immune microenvironment was changed, which might induce the formation and growth of the ectopic endometrium.

The lack of *MST1* expression in macrophages promotes the polarization of macrophages towards the M2 type and, at the same time, hypersecretion of IL-10. IL-10 as a cytokine, which could induce M2-type polarization of macrophages (44). IL-10 negatively controls inflammation, which could promote anti-inflammation and phagocytosis *via* MerTK/Gas6; IL-10/*STAT3* is one of the signaling pathway of anti-TNF agents downregulate cytokines associated with an anti-inflammatory phenotype in macrophages (45). In microglia, with the loss of autophagic flux, the expression of the M2-type marker IL-10 is inhibited, and it is also accompanied by abnormal expression of autophagy-related proteins, meanwhile, upregulation of autophagy by rapamycin promoted microglia polarization toward M2 phenotype (46). *ANXA1sp* potently increases IL-10 expression and also induces autophagic flux in the brain (47). Our study shows the increase of autophagy of ectopic endometrium may *via* the high level of IL-10 which secrets by endometriosis related peritoneal macrophages. The endometriosis related peritoneal macrophages towards to M2-type, manifested by hypersecretion of IL-10 and decreased phagocytosis, and the increase of CD163.

Ectopic endometriosis lesions have an unconventional level of autophagy, but the mechanism is unclear. Accordingly, we detected the expression of autophagic proteins using western blot and RT-qPCR, which showed increased expression of *Beclin1* and *LC3II/I*, and the decreased expression of *SQSTM1/p62* denoted the activation of autophagy. Multiplex tissue immunofluorescence showed that ectopic endometrial tissues had a strong fluorescence signal of *Beclin1* and *LC3*, and a weak fluorescence signal of *SQSTM1/p62*. Inhibition of autophagy-related genes weakens the ability of cell invasion and the strengthens the ability of cell migration in the late stage of cancer (48). Furthermore, we transfected the primary endometrial cells (ectopic and eutopic) with mCherry-GFP-*LC3* adenovirus and discovered that the process of fusion of lysosomes and autophagosomes to form autophagolysosomes was extremely smooth, indicating increased autophagy activation. Subsequently, we artificially knocked down *MST1* expression through transfection of miR-887-5p mimics or siRNA-*MST1* to transform M0 macrophages (THP-1+PMA), and then co-cultured them with HESCs to simulate the immune environment of ectopic endometriosis. After co-culture, HESCs showed high expression of *Beclin1* and *LC3II/I*, but decreased expression of *SQSTM1/p62*, and autophagolysosome formation increased according to the autophagy flux. These results indicate that endometriosis lesions have an increased autophagy state. As a selective autophagy adaptor protein, *SQSTM1/p62* plays a vital role in regulating nuclear transcription-related factor 2-antioxidant response elements, such as *NF-κB* and other signaling pathways and interacts with *LC3* to form autophagosomes (49, 50). The lack of *SQSTM1/p62* indicates increased formation of autophagosomes, which deplete *SQSTM1/p62*.

The activation of autophagy resulted in HESCs having a recycle model that may provide energy and a substance for the ectopic growth of endometrial cells. Restraining autophagy activation in ectopic endometrial cells might become an innovative approach for endometriosis treatment (51).

Autophagy is a type II programmed death, whereas apoptosis is a type I programmed death. When the cell is stimulated by certain internal and external factors, such as starvation and hypoxia, ATG13 anchors *ULK1* to the pre-autophagy body structure (PAS), and then most autophagy-related (Atg) proteins are aggregated into PAS in stages, marking the beginning of autophagy. Subsequently, the *ULK1* complex, PI3K complex (*Beclin1* is its subunit), ATG9A system, ATG12 coupling system, and *LC3* coupling system hierarchically target PAS nucleation, coupled with the elongation of the separation membrane to form mature autophagosomes. Atg12-Atg5 and Atg8/*LC3* coupling systems mediate the formation of a closed bilayer membrane structure, and mature autophagosomes fuse with lysosomes in the perinuclear region to form autophagic lysosomes (52–56). Immediately, several enzymes (including lysosomal hydrolases) in the original lysosomes affect autophagosomes and cytoplasmic substances, such as proteins and organelles in the inner membrane, degrading them into amino acids or peptides for reuse by cells (57–60). There are three types of autophagy with different morphologies and mechanisms in cells: macroautophagy, microautophagy, and molecular chaperone-mediated autophagy. Macroautophagy is usually called autophagy. Here, cytoplasmic macromolecules, aggregate proteins, damaged organelles, or pathogens are transported to lysosomes to form autophagic lysosomes, which are digested by lysosomal hydrolase to produce nucleotides, amino acids, fatty acids, sugars, and ATP, and finally, circulate into the cytoplasm. The self-digestion of cells mediated by lysosomes maintains cell metabolism and survival under starvation and stress, thus removing damaged proteins and organelles to maintain the quality and quantity of proteins and organelles. Autophagy plays a pathophysiological role in many disease processes, including cancer, neurodegeneration, autoimmune diseases, aging, cell death, heart disease, and infections, and helps cells remove damaged proteins, organelles, pathogens, or aggregates (61–65). Therefore, as autophagy-related proteins that are detected in the entire autophagy process, we selected *Beclin1*, *LC3*, and p62 as indicators to evaluate autophagy levels through semi-quantitative detection of protein and mRNA levels and localization by immunofluorescence and immunohistochemistry. In this study, autophagy in endometriotic lesions was abnormally activated, and the autophagy level was similar to that of malignant tumor cells.

Our findings show that in the pro-inflammatory peritoneal immune microenvironment of endometriosis patients, the frequency of both the innate and adaptive immune systems was changed, and macrophages were the most significant. Moreover, the abnormal expression of *MST1* in endometriosis-related peritoneal macrophages could activate autophagy in ectopic endometrial lesions. Using mCherry-GFP-*LC3* intuitively revealed increased autophagy and confirmed that *MST1*-deficient macrophages may experience this phenomenon. *MST1*-deficient macrophages provide an opportunity for the activation and accumulation of *p38-MAPK*, which plays a striking role in macrophage polarization. This study focused on the reprogramming of peritoneal macrophages *via* inducing *MST1* deficiency and increasing *p38-MAPK* expression, which led to an increase in the anti-inflammatory properties of macrophages and promotion of tissue repair, creating higher levels of autophagy in endometriosis lesions. Similar conditions of endometrial carcinoma and ovarian cancer also provide a new perspective for the study of the mechanism underlying malignant transformation of endometriosis. Abnormal autophagy may induce epithelial-mesenchymal transition (EMT) (66), which contributes to the invasion and metastasis of cancers, indicating the probable mechanism of ectopic growth of endometrial cells. Studies have reported that inhibition of autophagy could induce EMT *via* ROS/*HO-1* in ovarian cancer (67). Data from our clinical patients and *in vitro* experiments supplement our knowledge concerning innate immune cell regulation in endometriosis, particularly macrophages, and put forward the details regarding the existence of a hub between macrophages and endometrial cells.

These features of endometriosis-associated macrophages are closely related to secretion, polarization, and phagocytosis (29, 30). The M1-type macrophages are activated by LPS (Lipopolysaccharide), and M2-type macrophages, which are activated by IL-4, can transform reciprocally *in vivo*, while recent studies have suggested that these types of macrophages are dynamic (31–33). M2-type macrophages have anti-inflammatory and anti-cancer functions; but in endometriosis, M2-type macrophages may be pathogenic, the CIBERSORT and WGCNA algorithms revealed that M2 macrophages were related to endometriosis (38). Our previous study demonstrated that reprogramming of M2 macrophages may inhibit the development of endometriosis M1NVs suppressed the development of endometriosis through reprogramming of M2 macrophages (39).

All of this work was done *in vitro*, and we used patient lesions and peritoneal cells to discover the phenomenon, with subsequent validation in cell lines and primary cells.

## Data availability statement

The data used to support the findings of this study are available from the corresponding author upon request.

## Ethics statement

The studies involving human participants were reviewed and approved by Institutional Review Board of Qilu Hospital of Shandong university KYLL-2020(KS)-177. The patients/participants provided their written informed consent to participate in this study. Written informed consent was obtained from the individual(s) for the publication of any potentially identifiable images or data included in this article.

## Author contributions

YH, SY, XD, XJ, SW, DL and GW participated in the design of this research. YH, XJ, SW, and GW collected the clinical patients’ specimens. YH, SY, and XD finished the related experiments. YH processed and statistically analyze data and write manuscript. GW revised this manuscript. All authors contributed to the article and approved the submitted version.

## Funding

This study was supported by the National Natural Science Foundation of China [grant numbers 82071621, and 81901458], the Major Program of Shandong Provincial Natural Science Foundation [ZR2021ZD34], the Key Technology Research and Development Program of Shandong [grant number 2019GSF108071].

## Acknowledgments

We thank Translational Medicine Core Facility of Shandong University for consultation and instrument availability that supported this work.

## Conflict of interest

The authors declare that the research was conducted in the absence of any commercial or financial relationships that could be construed as a potential conflict of interest.

## Publisher’s note

All claims expressed in this article are solely those of the authors and do not necessarily represent those of their affiliated organizations, or those of the publisher, the editors and the reviewers. Any product that may be evaluated in this article, or claim that may be made by its manufacturer, is not guaranteed or endorsed by the publisher.

## Abbreviations

EMT, epithelial-mesenchymal transition; HESCs, human endometrial storm cell; FBS, fetal bovine serum; *MAPK*, mitogen-activated protein kinase; *MST1*, mammalian sterile 20-like kinase 1; NC, negative control; LPS, Lipopolysaccharide; PMA, Phorbol 12-Myristate 13-Acetate; pMφ, peritoneal macrophages; PS, penicillin-streptomycin; RT-qPCR, Quantitative real-time PCR.
