# Supplementary figures and images for: Deficiency of MST1 in endometriosis related peritoneal macrophages promoted the autophagy of ectopic endometrial stromal cells by IL-10
Source: Front Immunol. 2022 Oct 3;13:993788. doi: 10.3389/fimmu.2022.993788 (PMC9575673; doi:10.3389/fimmu.2022.993788)

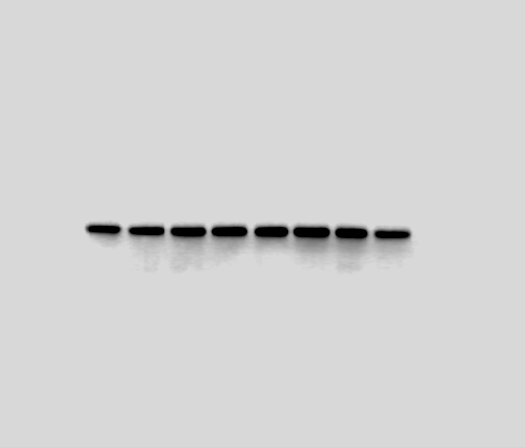

Supplement: Supplementary file 1 [file DataSheet_1.zip › The original image of western blot/Fig1 western blot/fig1C-GAPDH.tif]

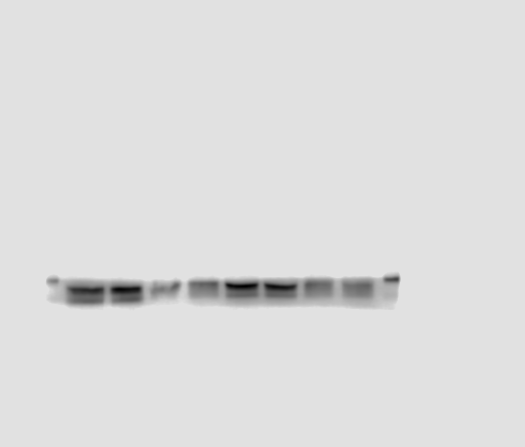

Supplement: Supplementary file 1 [file DataSheet_1.zip › The original image of western blot/Fig1 western blot/fig1C-MAPK.tif]

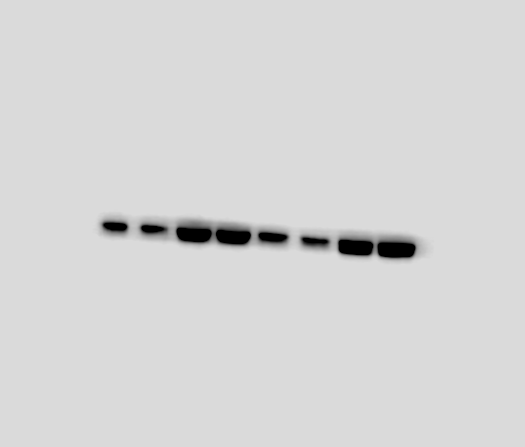

Supplement: Supplementary file 1 [file DataSheet_1.zip › The original image of western blot/Fig1 western blot/fig1C-MST1.tif]

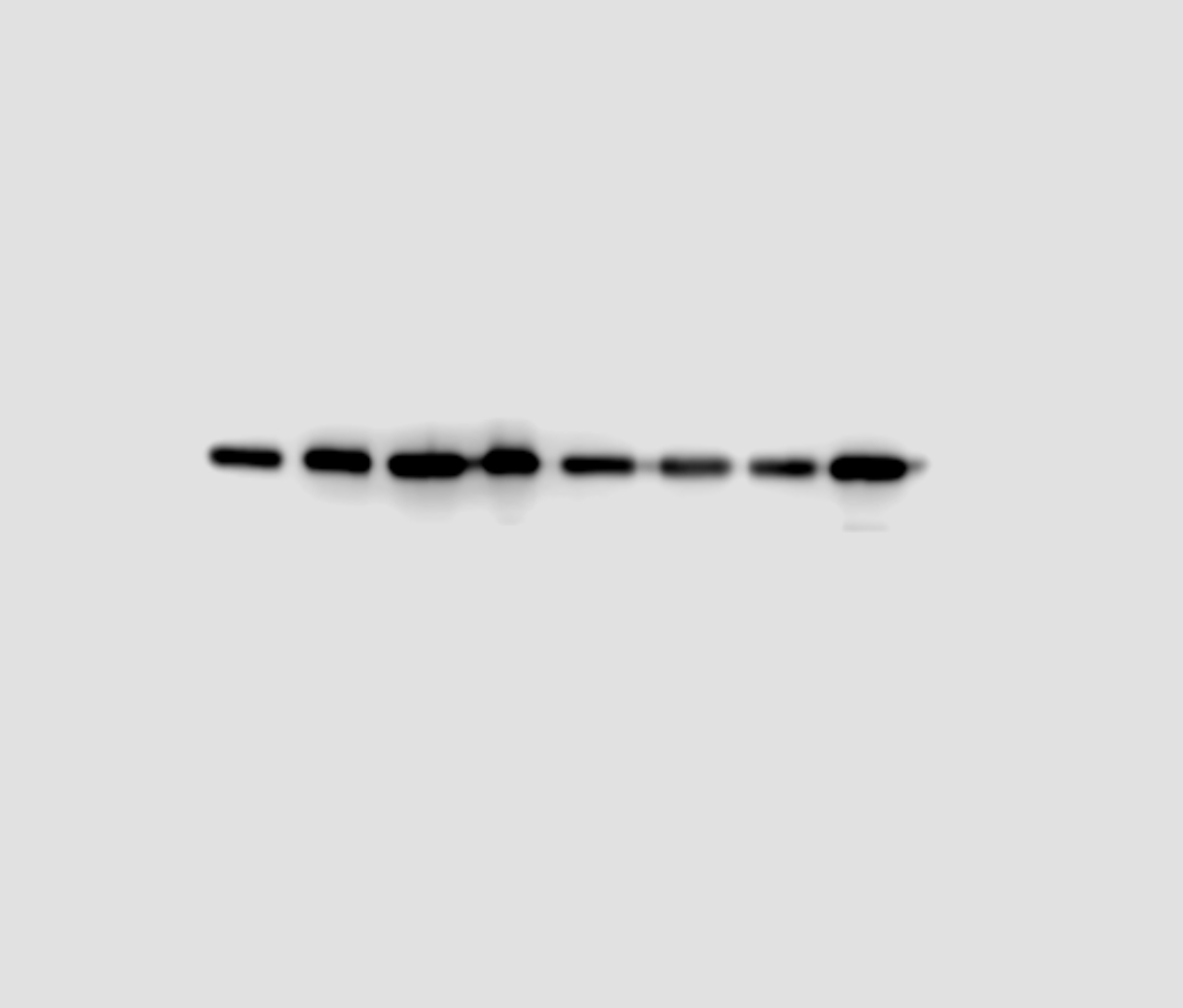

Supplement: Supplementary file 1 [file DataSheet_1.zip › The original image of western blot/Fig2 western blot/fig2. F -MST1.jpg]

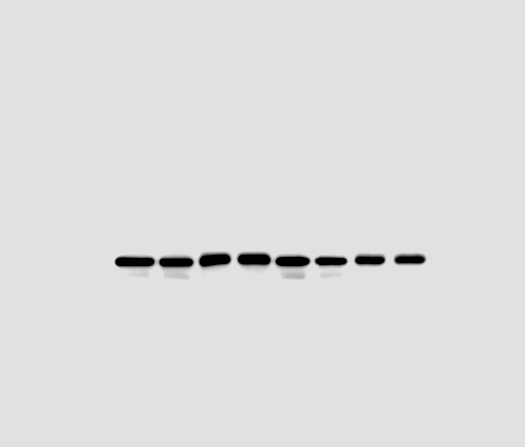

Supplement: Supplementary file 1 [file DataSheet_1.zip › The original image of western blot/Fig2 western blot/fig2.F-GAPDH.tif]

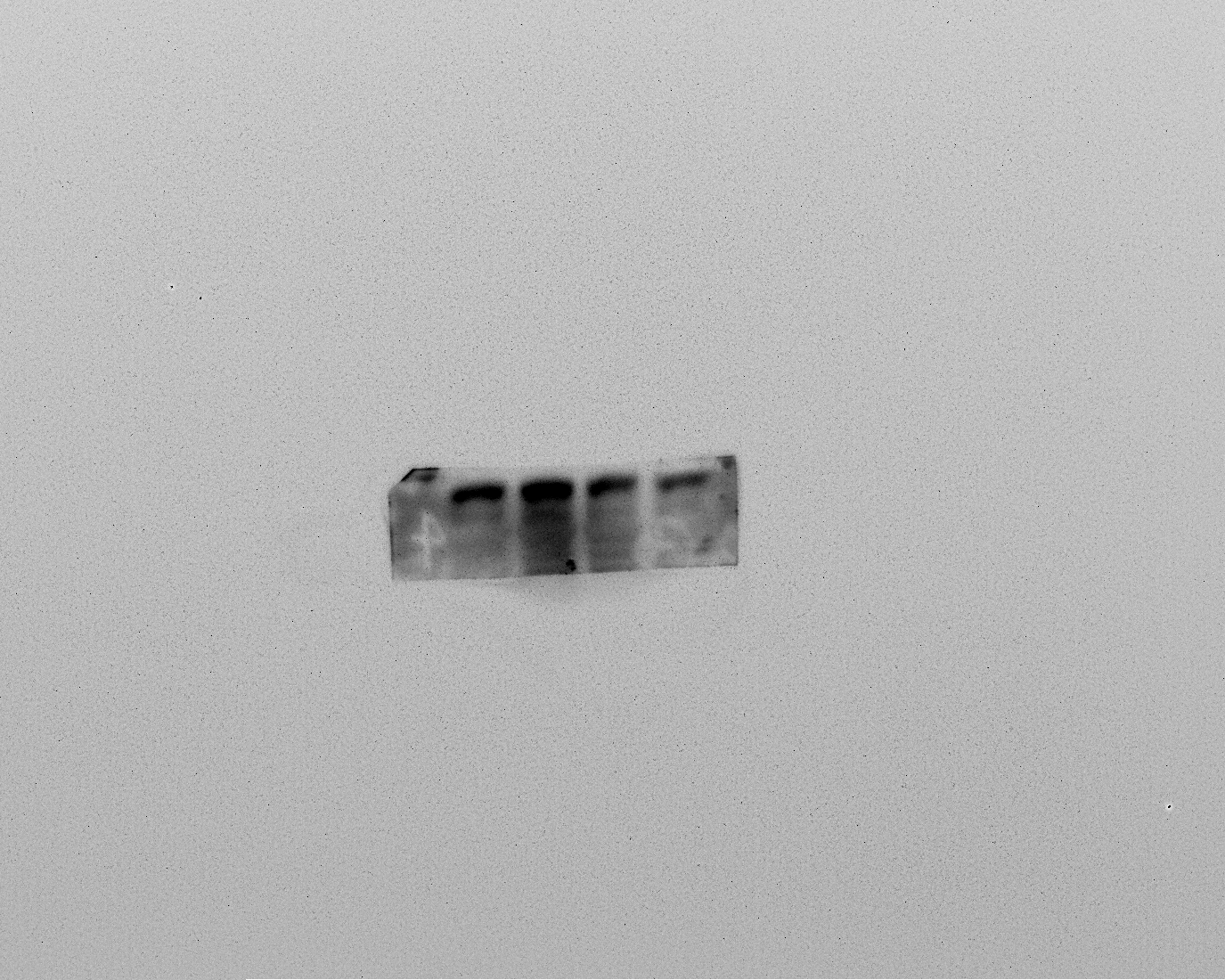

Supplement: Supplementary file 1 [file DataSheet_1.zip › The original image of western blot/Fig2 western blot/fig2.F-MAPK.tif]

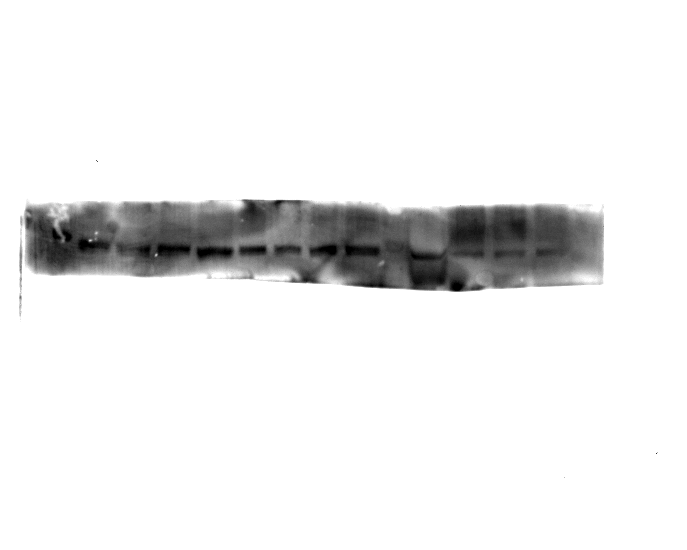

Supplement: Supplementary file 1 [file DataSheet_1.zip › The original image of western blot/Fig3 western blot/fig3. A-Beclin1.tif]

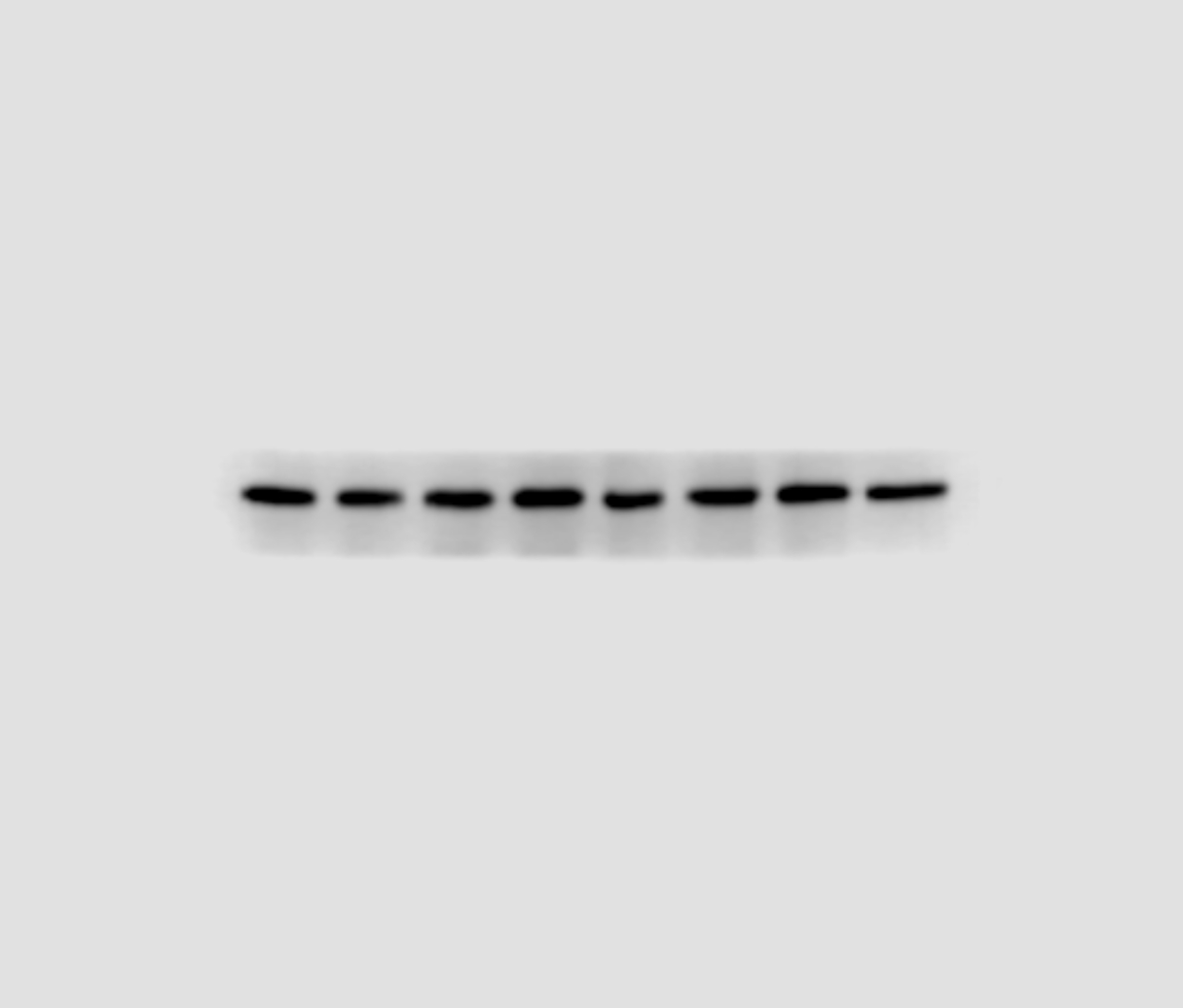

Supplement: Supplementary file 1 [file DataSheet_1.zip › The original image of western blot/Fig3 western blot/fig3. A-GAPDH.jpg]

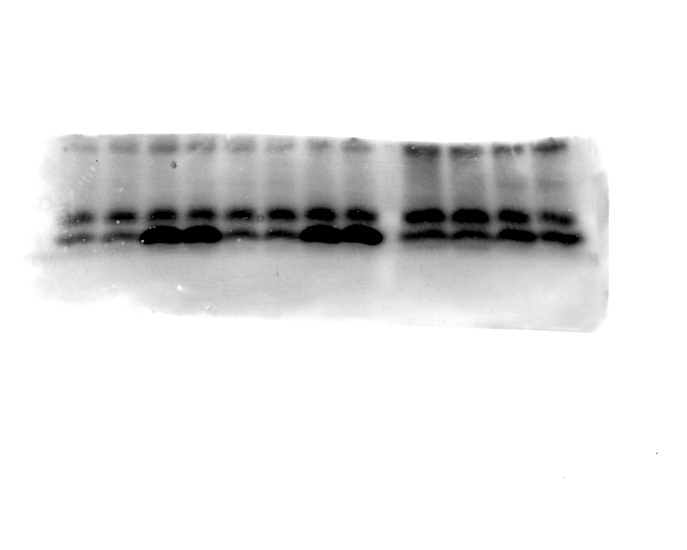

Supplement: Supplementary file 1 [file DataSheet_1.zip › The original image of western blot/Fig3 western blot/fig3. A-LC3.tif]

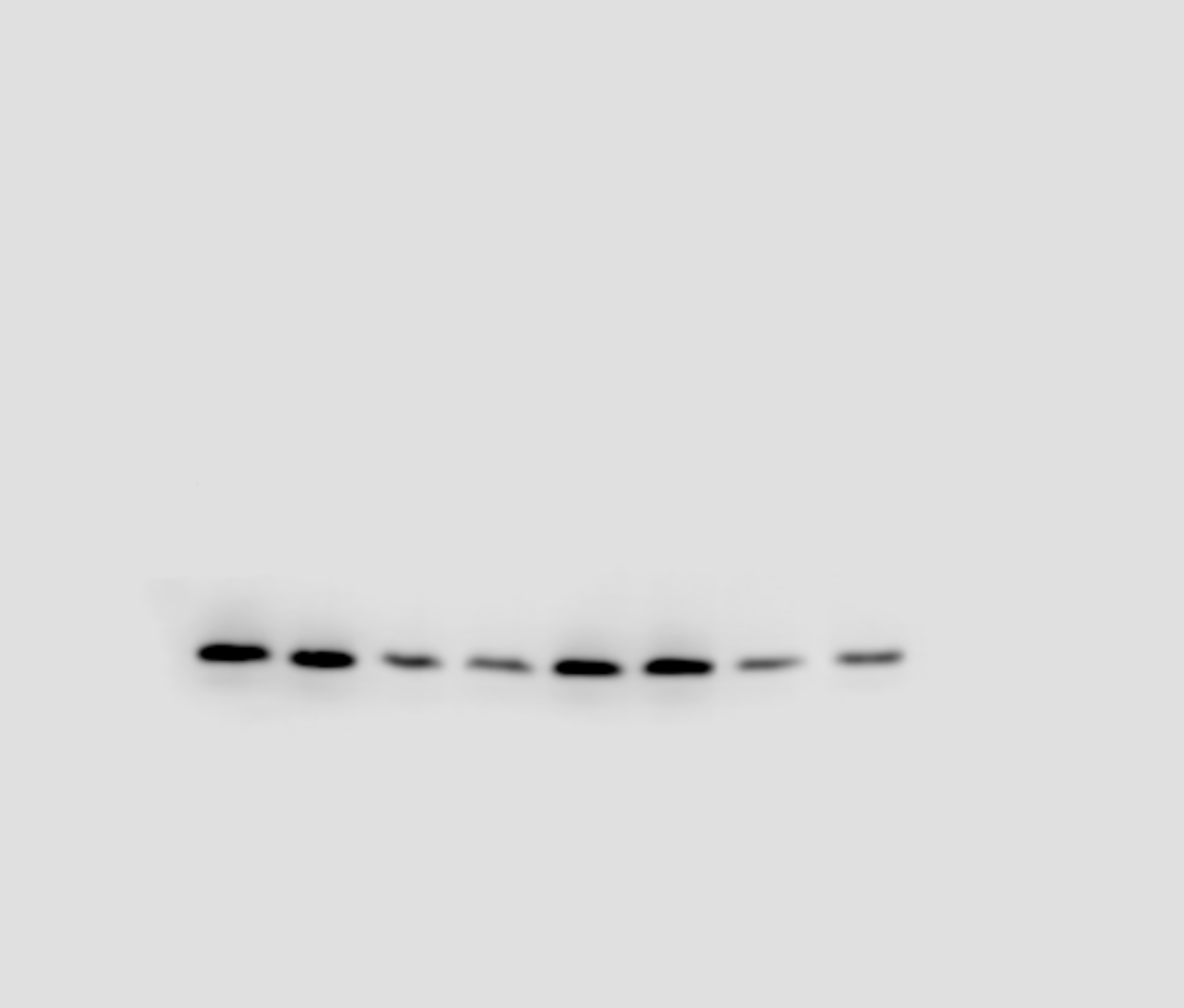

Supplement: Supplementary file 1 [file DataSheet_1.zip › The original image of western blot/Fig3 western blot/fig3. A-SQSTM1p62.tif]

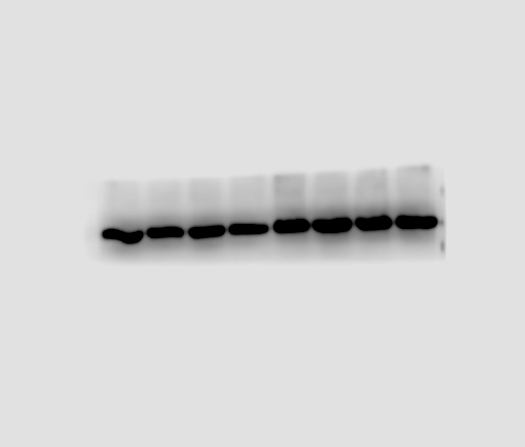

Supplement: Supplementary file 1 [file DataSheet_1.zip › The original image of western blot/Fig4 western blot/fig4.A-GAPDH.tif]

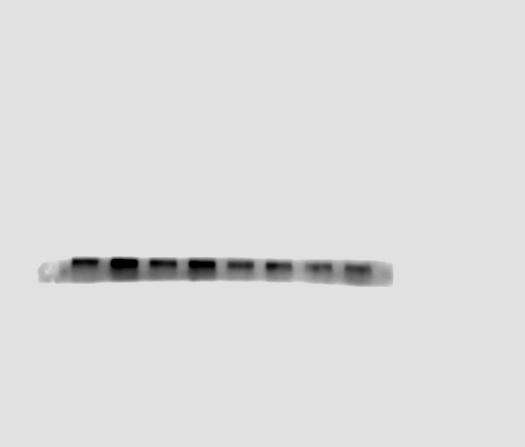

Supplement: Supplementary file 1 [file DataSheet_1.zip › The original image of western blot/Fig4 western blot/fig4.A-MST1.tif]

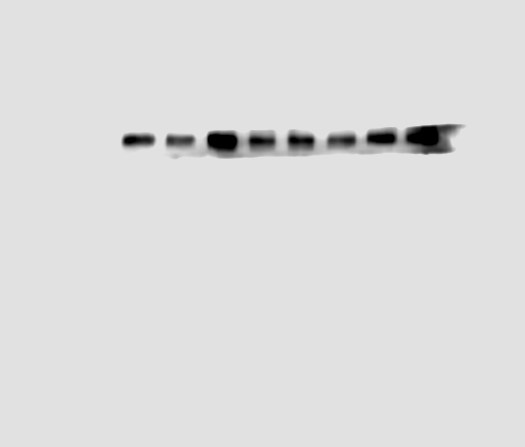

Supplement: Supplementary file 1 [file DataSheet_1.zip › The original image of western blot/Fig4 western blot/fig4.A-p38-MAPK.tif]

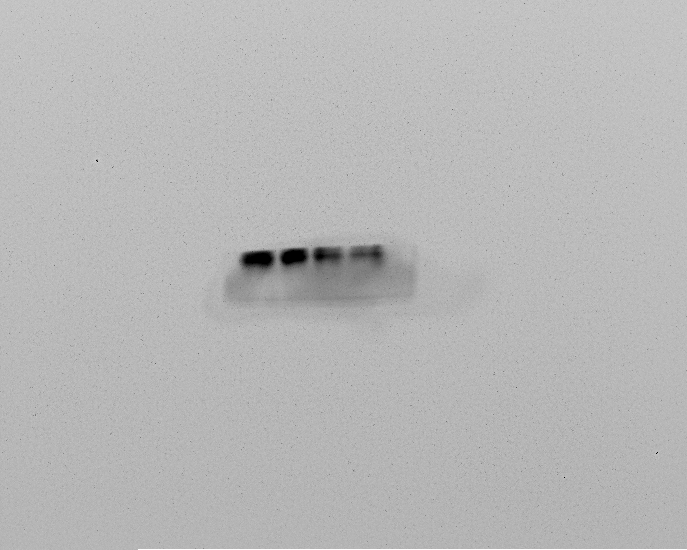

Supplement: Supplementary file 1 [file DataSheet_1.zip › The original image of western blot/Fig5 western blot/fig5. C-SQSTM1p62.tif]

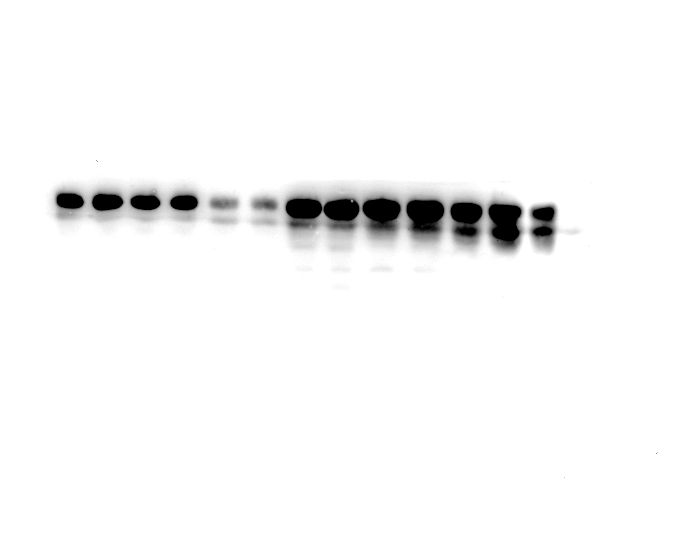

Supplement: Supplementary file 1 [file DataSheet_1.zip › The original image of western blot/Fig5 western blot/fig5.C GAPDH.tif]

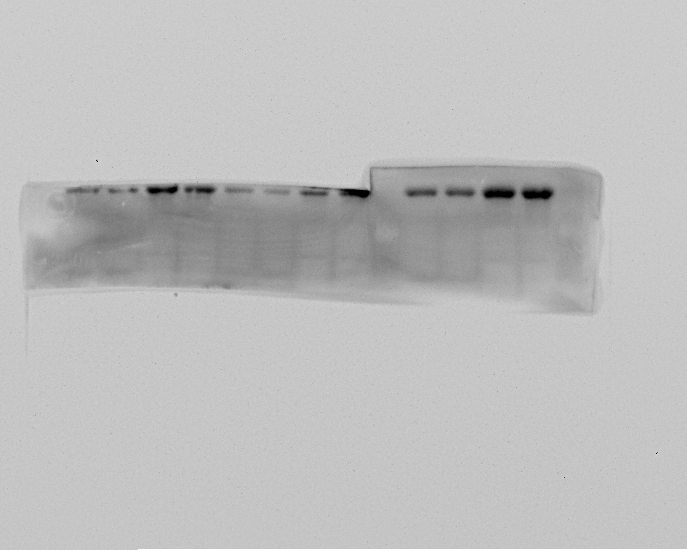

Supplement: Supplementary file 1 [file DataSheet_1.zip › The original image of western blot/Fig5 western blot/fig5.C-Beclin1.tif]

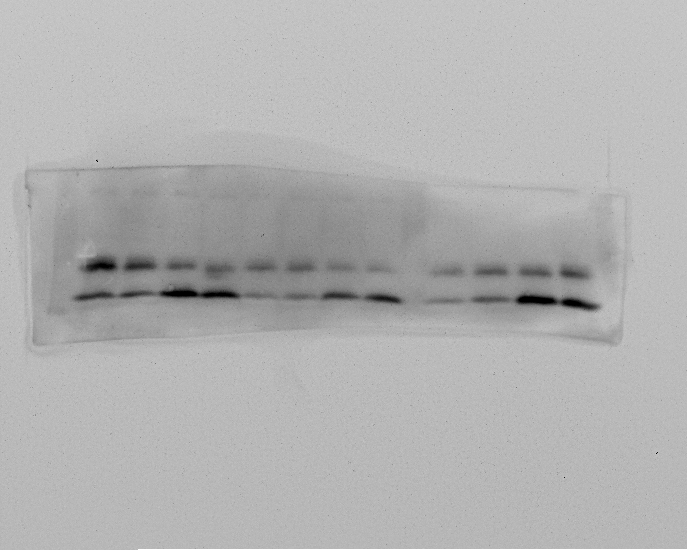

Supplement: Supplementary file 1 [file DataSheet_1.zip › The original image of western blot/Fig5 western blot/fig5.C-LC3.tif]

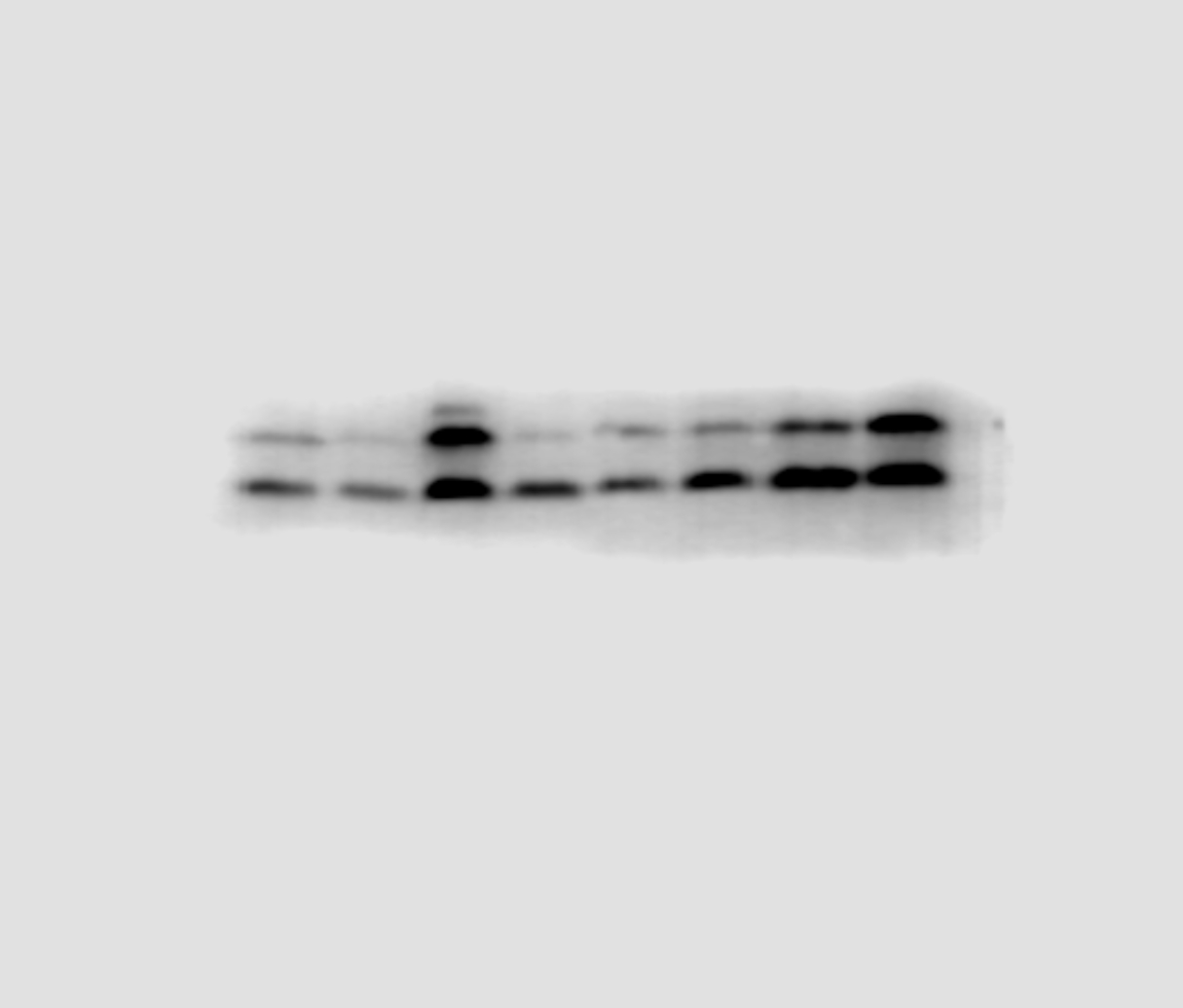

Supplement: Supplementary file 1 [file DataSheet_1.zip › The original image of western blot/Fig6 western blot/fig6. A-LC3.jpg]

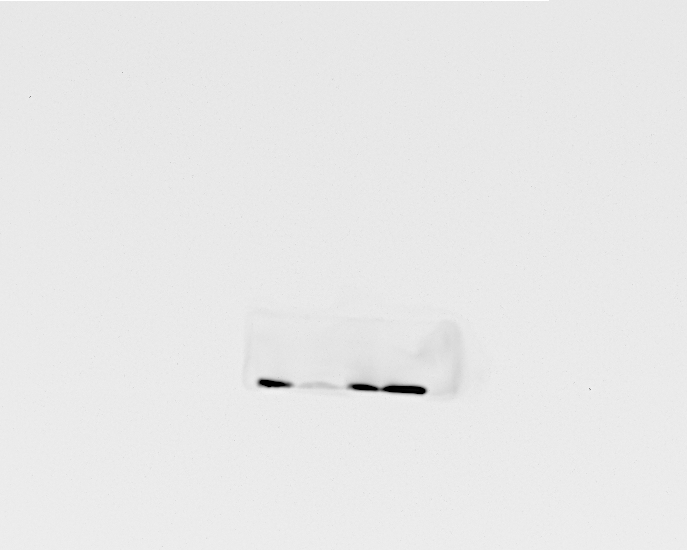

Supplement: Supplementary file 1 [file DataSheet_1.zip › The original image of western blot/Fig6 western blot/fig6. A-SQSTM1p62.tif]

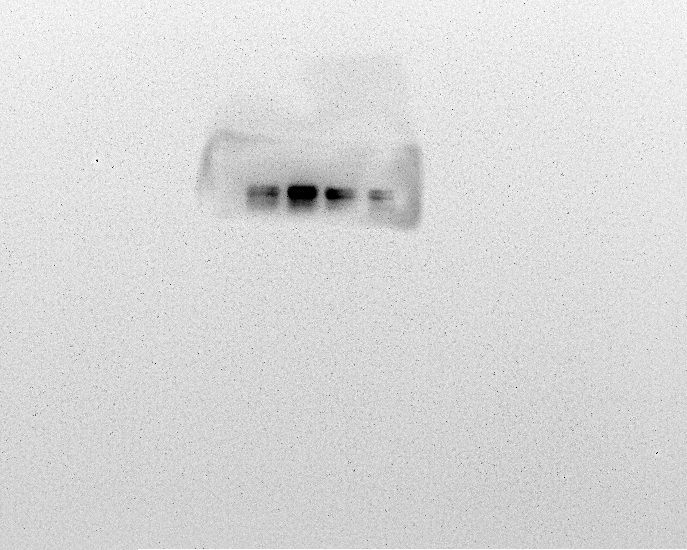

Supplement: Supplementary file 1 [file DataSheet_1.zip › The original image of western blot/Fig6 western blot/fig6.A-Beclin1.tif]

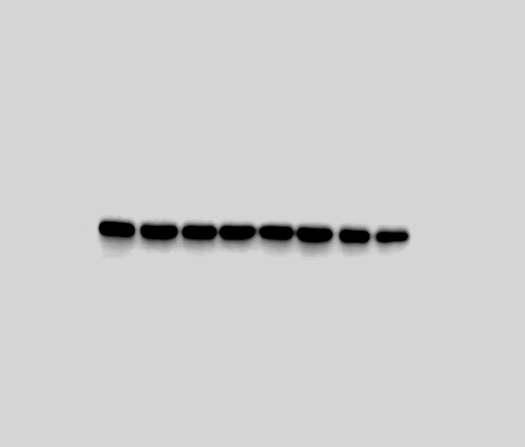

Supplement: Supplementary file 1 [file DataSheet_1.zip › The original image of western blot/Fig6 western blot/fig6.A-GAPDH.tif]
